# Obesity and Impaired Metabolic Health Increase Risk of COVID-19-Related Mortality in Young and Middle-Aged Adults to the Level Observed in Older People: The LEOSS Registry

**DOI:** 10.3389/fmed.2022.875430

**Published:** 2022-05-11

**Authors:** Norbert Stefan, Katrin Sippel, Martin Heni, Andreas Fritsche, Robert Wagner, Carolin E. M. Jakob, Hubert Preißl, Alexander von Werder, Yascha Khodamoradi, Stefan Borgmann, Maria Madeleine Rüthrich, Frank Hanses, Martina Haselberger, Christiane Piepel, Martin Hower, Jürgen vom Dahl, Kai Wille, Christoph Römmele, Janne Vehreschild, Melanie Stecher, Michele Solimena, Michael Roden, Annette Schürmann, Baptist Gallwitz, Martin Hrabe de Angelis, David S. Ludwig, Matthias B. Schulze, Bjoern Erik Ole Jensen, Andreas L. Birkenfeld

**Affiliations:** ^1^Institute of Diabetes Research and Metabolic Diseases (IDM) of the Helmholtz Center Munich, Tübingen, Germany; ^2^Department of Internal Medicine IV, University Hospital of Tübingen, Tübingen, Germany; ^3^German Center for Diabetes Research (DZD), Munich, Germany; ^4^Department of Internal Medicine I, Faculty of Medicine, University Hospital Cologne, Cologne, University of Cologne, Cologne, Germany; ^5^German Center for Infection Research (DZIF), Partner-Site Bonn-Cologne, Cologne, Germany; ^6^Department of Internal Medicine II, School of Medicine, University Hospital Rechts der Isar, Technical University of Munich, Munich, Germany; ^7^Department of Internal Medicine, Infectious Diseases, University Hospital Frankfurt, Goethe University Frankfurt, Frankfurt am Main, Germany; ^8^Department of Infectious Diseases and Infection Control, Ingolstadt Hospital, Ingolstadt, Germany; ^9^Department of Internal Medicine II, University Hospital Jena, Jena, Germany; ^10^Emergency Department, University Hospital Regensburg, Regensburg, Germany; ^11^Department of Internal Medicine I, Passau Hospital, Passau, Germany; ^12^Department of Internal Medicine I, Hospital Bremen-Center, Bremen, Germany; ^13^Department for Pneumology, Infectiology, Internal Medicine and Intensive Care, gGmbH, Dortmund, Germany; ^14^Division of Cardiology, Hospital Maria Hilf Mönchengladbach, Mönchengladbach, Germany; ^15^University Clinic for Hematology, Oncology, Hemostaseology and Palliative Care, University of Bochum, Minden, Germany; ^16^Internal Medicine III - Gastroenterology and Infectious Diseases, University Hospital of Augsburg, Augsburg, Germany; ^17^Department of Internal Medicine, Hematology and Oncology, University Hospital Cologne, Goethe University Frankfurt, Frankfurt am Main, Germany; ^18^Helmholtz Center Munich, Faculty of Medicine, Paul Langerhans Institute Dresden, University Hospital, Carl Gustav Carus, Technische Universität Dresden, Dresden, Germany; ^19^Department of Endocrinology and Diabetology, Medical Faculty and University Hospital, Heinrich-Heine University, Düsseldorf, Germany; ^20^Institute for Clinical Diabetology, German Diabetes Center, Leibniz Center for Diabetes Research at Heinrich- Heine University, Düsseldorf, Germany; ^21^Department of Experimental Diabetology, German Institute of Human Nutrition Potsdam-Rehbruecke, Nuthetal, Germany; ^22^Institute of Experimental Genetics, Helmholtz Zentrum München, Oberschleißheim, Germany; ^23^TUM School of Life Sciences (SoLS), Chair of Experimental Genetics, Technische Universität München, Freising, Germany; ^24^New Balance Foundation Obesity Prevention Center, Boston Children's Hospital, Boston, MA, United States; ^25^Department of Pediatrics, Harvard Medical School, Boston, MA, United States; ^26^Department of Nutrition, Harvard T.H. Chan School of Public Health, Boston, MA, United States; ^27^Department of Molecular Epidemiology, German Institute of Human Nutrition Potsdam-Rehbruecke, Nuthetal, Germany; ^28^Department of Gastroenterology, Hepatology and Infectious Diseases, Medical Faculty and University Hospital Düsseldorf, Heinrich Heine University Düsseldorf, Düsseldorf, Germany

**Keywords:** obesity, diabetes, hypertension, impaired metabolic health, mortality, COVID-19

## Abstract

Advanced age, followed by male sex, by far poses the greatest risk for severe COVID-19. An unresolved question is the extent to which modifiable comorbidities increase the risk of COVID-19-related mortality among younger patients, in whom COVID-19-related hospitalization strongly increased in 2021. A total of 3,163 patients with SARS-COV-2 diagnosis in the Lean European Open Survey on SARS-CoV-2-Infected Patients (LEOSS) cohort were studied. LEOSS is a European non-interventional multi-center cohort study established in March 2020 to investigate the epidemiology and clinical course of SARS-CoV-2 infection. Data from hospitalized patients and those who received ambulatory care, with a positive SARS-CoV-2 test, were included in the study. An additive effect of obesity, diabetes and hypertension on the risk of mortality was observed, which was particularly strong in young and middle-aged patients. Compared to young and middle-aged (18–55 years) patients without obesity, diabetes and hypertension (non-obese and metabolically healthy; *n* = 593), young and middle-aged adult patients with all three risk parameters (obese and metabolically unhealthy; *n* = 31) had a similar adjusted increased risk of mortality [OR 7.42 (95% CI 1.55–27.3)] as older (56–75 years) non-obese and metabolically healthy patients [*n* = 339; OR 8.21 (95% CI 4.10–18.3)]. Furthermore, increased CRP levels explained part of the elevated risk of COVID-19-related mortality with age, specifically in the absence of obesity and impaired metabolic health. In conclusion, the modifiable risk factors obesity, diabetes and hypertension increase the risk of COVID-19-related mortality in young and middle-aged patients to the level of risk observed in advanced age.

## Introduction

As of 14 February 2022, more than 404 million people worldwide have been infected with SARS-CoV-2, resulting in more than 5.7 million deaths (1). Early in the SARS-CoV-2 pandemic, older age was identified as the strongest risk factor for COVID-19-related mortality. Furthermore, male sex and several comorbidities were found to be associated with an increased risk of mortality in patients with COVID-19 (2–4). Obesity and hyperglycemia in the non-diabetic range were additionally identified as potential risk factors for COVID-19 morbidity and mortality (5–9). Of note, these relationships were independent of age, sex and other comorbidities (10–14). Consequently, obesity and impaired metabolic health are now viewed as important modifiable risk factors for disease severity (15–17).

However, recently, in a large, international, multicenter study from 18 sites in 11 countries, of 7,244 patients hospitalized with COVID-19, obesity and diabetes were found to associate with increased adjusted odds of supplemental oxygen/non-invasive ventilatory support, yet, not with mortality (18). Furthermore, in a very large community-based cohort study from the United Kingdom that evaluated data from 6,910,695 patients with a positive SARS-CoV-2 test result, obesity strongly associated with mortality in the younger and middle-aged adults, but not in the older patients (19). Unfortunately, in that study no adjustment for comorbidities could be done. Thus, it is important to clarify whether obesity and other metabolic comorbidities may increase the risk of COVID-19-related mortality, independently of other diseases, specifically in younger and middle-aged patients.

These patients with COVID-19 are generally considered to have substantially lower risk of COVID-19-related mortality, than those older than 65 years. However, risk in younger age groups has become increasingly relevant, with initially selective vaccination of older individuals and rapidly rising incidence of infection and hospitalization among children, adolescents, and young adults (20). Data from the US Centers for Disease Control and Prevention (CDC) suggests that a 35-year-old with diabetes mellitus, hypertension, cardiovascular disease, obesity, or other chronic conditions had a similar risk of COVID-19-related death as a 65-year-old with none of these conditions (21). Furthermore, in an analysis of data from an US Premier Healthcare Database of hospital-based patients with COVID-19, younger patients (age 18–34 years) with morbid obesity, hypertension, and diabetes faced similar risk of death or need for mechanical ventilation, as that observed in middle-aged (age 35–64 years) adults (22). However, these did not consider potential confounding, and in the CDC report no information about comorbidities was available in 22% of the patients (21). Adjustment for sex and other comorbidities, such as cardiovascular, renal and liver disease, is essential, as these comorbidities are strongly related to impaired metabolic health.

To clarify the potential impact of obesity and impaired metabolic health on COVID-19 related mortality in younger adults, we have studied the determinants of COVID-19-related mortality in 3,163 patients with COVID-19 of the Lean European Open Survey on SARS-CoV-2-Infected Patients (LEOSS) cohort study.

## Research Design and Methods

### Study Design and Patient Cohort

A total of 6,457 consecutive patients, who were included in the LEOSS registry between March 2020 and February 2021, were evaluated. LEOSS is a European non-interventional multi-center cohort study established in March 2020 to address the lack of information on the epidemiology and clinical course of SARS-CoV-2 infection (23, 24). The registry collects data on hospitalized patients of all ages and patients who receive ambulatory medical consultation. As of July 2020, more than 125 sites from 7 different countries have been registered to LEOSS. Daily statistics are provided on the LEOSS website (https://leoss.net). To facilitate the rapid data acquisition needed during a pandemic, LEOSS involves autonomous, self-managed study sites that collect data in an anonymous form. To achieve this, no directly identifying data are stored in the registry and demographic data as well as timestamps are only collected in a rough form. Furthermore, data were documented categorically. Patient privacy was additional protected using the anonymization procedures described by Jakob et al. (24). Data collection is performed once per case, retrospectively after treatment has finished or the patient has died. Although this method precludes longitudinal data collection and follow-up of discharged patients, it has the advantage that no informed consent is necessary. Furthermore, this method provides for the inclusion of data on children and unconscious or deceased patients and avoids problems that could arise from language barriers. All patients had a diagnosis confirmed by positive results of PCR testing. Approval for LEOSS was obtained by the applicable local ethics committees of all participating centers and registered at the German Clinical Trails Register (DRKS, No. S00021145).

### Clinical Data and Outcomes

Data were recorded in an electronic case report form operated using the online cohort platform ClinicalSurveys.net, which was developed by the University Hospital of Cologne (UHC), Germany. ClinicalSurveys.net was hosted by QuestBack, Oslo, Norway on servers of UHC, Cologne, as part of a software-as-a-service agreement. Baseline data closest to the first positive SARS-CoV-2 test were analyzed. Demographic, clinical, laboratory and outcome data were extracted from the in-hospital medical records. Operational definitions of the co-morbidities studied are based on the medical diagnosis guidelines that were applied by the treating physicians in the hospital. Diagnosis were either pre-known or newly made by the treating physicians based on the clinical in-hospital evaluation and/or laboratory results. Analyzed laboratory data were collected within 48 h of a positive SARS-CoV-2 PCR result, irrespective of the patient's status. Among the 6,457 patients evaluated only adult (age ≥18 years) patients who had complete information about sex, age, BMI and the comorbidities diabetes, hypertension, coronary artery disease, chronic kidney disease and chronic liver disease (*N* = 3,517) were considered eligible for the analyses. Among them, a total of 354 patients with missing information on survival were excluded, yielding a sample of 3,163 for the main analyses (Supplementary Figure 1).

Comorbidities were dichotomized (e.g., diabetes present/absent, coronary artery disease present/absent). Comorbidities were set to unknown/missing when all specific comorbidities of one group were unknown or missing. Values documented as unknown were defined as missing. Besides sex, age, BMI and the above-mentioned comorbidities, the following clinical parameters related to metabolic risk, which were not available in all patients, were evaluated: hemoglobin A1c (HbA1c), serum creatinine, serum C-reactive protein (CRP), serum interleukin-6 (IL-6), serum alanine aminotransferase (ALT), serum aspartate aminotransferase (AST), serum gamma-glutamyl transferase (GGT), as well as urine ketone bodies. Clinical parameters were set to unknown/missing if not available. The primary outcome was COVID-19-related mortality. In an exploratory approach disease severity, which is not a hard endpoint, was also studied (uncomplicated phase: patients were either asymptomatic, and had symptoms of upper respiratory tract infection, fever or nausea, emesis, or diarrhea; complicated phase: patients had at least one of the characteristics new need for oxygen supplementation or clinically relevant increase of prior oxygen home therapy, PaO2 at room air < 70 mmHg, SO2 at room air < 90%, increase of AST or ALT > 5 × upper limit of normal, new cardiac arrythmia, new pericardial effusion > 1 cm or new heart failure with pulmonary edema, congestive hepatopathy, or peripheral edema; critical phase patients were dependent on catecholamines, experienced life-threatening cardiac arrhythmia, had mechanical ventilation (invasive or non-invasive), or need for unplanned mechanical ventilation prolongation (> 24 h) of planned mechanical ventilation, liver failure with an INR > 3.5 (quick < 50%), a qSOFA score of > = 2, or acute renal failure with need of dialysis).

### Statistical Analyses

We calculated and report patient characteristics as absolute numbers and percentages. For comparison of percentages between groups the χ^2^-test was used. The odds ratios of baseline characteristics, comorbidities and laboratory parameters, with mortality were assessed in univariate and in multivariable logistic regression models. Univariate and multivariable relationships of baseline characteristics with mortality were also assessed after patients were stratified in young and middle aged (18–55 years; *n* = 1,068), older age (56–75 years; *n* = 1,220) and old age (>75 years; *n* = 875) groups. Then patients in each age group were further categorized by the presence or absence of obesity, of obesity+diabetes and of obesity+diabetes+hypertension. For the main analyses, patients in the three age groups were subdivided into those (i) without obesity (BMI <30 kg·m^−2^) and without impaired metabolic health (no diabetes and no hypertension, *n* = 1,098) and in those (ii) having all three risk factors (BMI ≥30 kg·m^−2^, diabetes and hypertension, *n* = 259). Kaplan-Meier analyses were used to compare the survival of the patients among these six subgroups. A *p* < 0.05 was considered to indicate statistical significance. Data management, statistical analysis, and computation of figures were conducted using R (R Development Core Team, Vienna, Austria, Version 3.5.2., 2019). Additional information about the LEOSS questionnaire can be found under https://leoss.net/.

## Results

Among the 3,163 patients included in the analyses, data were collected primarily from Germany (*N* = 95%), as well as from Turkey, Belgium, Switzerland, Spain, Austria, Italy, Bosnia and Herzegovina, United Kingdom and Latvia. A total of 2,989 from 3,144 patients (19 patients with missing information) had an inpatient stay. Disease course was classified as uncomplicated (*N* = 1,284) complicated (*N* = 1,130) and critical (*N* = 749) (24). From the 3,163 patients studied, 2,661 patients recovered from the disease while 502 patients died (Supplementary Table 1).

### Univariable and Multivariable Relationships of Patient Characteristics With Mortality

In univariable analyses, among the parameters age, sex, BMI, comorbidities and selected laboratory variables, determined at the day of SARS-COV-2 diagnosis, higher age, male sex, diabetes, hypertension, HbA1c >10%, coronary artery disease, chronic liver disease and liver cirrhosis were associated with an increased risk of mortality (Supplementary Table 2). In a multivariable regression model including all studied parameters, higher age, male sex, BMI ≥35 kg·m^−2^, diabetes, HbA1c >8.1%, CRP ≥30 mg/L and GGT >10 upper limit of normal were independently associated with an increased risk of mortality (Supplementary Table 3).

To avoid over-adjustment in the statistical models by including variables that are highly related to each other, e.g., the diagnosis of liver cirrhosis and elevated transaminases or chronic kidney disease and elevated serum creatinine, we further focused in the multivariate regression models on the parameters reported in the Table 1. In that parsimonious multivariable regression model higher age, male sex, BMI ≥ 35 kg·m^−2^, HbA1c >10%, chronic kidney disease and liver cirrhosis were independently associated with an increased risk of mortality. The association with hypertension was borderline, with an adjusted *p*-value of 0.056 (Table 1, Supplementary Figure 2).

**Table 1:** Multivariable relationships of selected anthropometrics, comorbidities and laboratory parameters with COVID-19-related mortality.

**Characteristics**	**Recovered/died**	**OR**	**Lower 95%CI**	**Upper 95%CI**	***p***
Age 18–25 (years)	71/0	0.00	0.000	0.00	0.97
Age 26–35 (years) (ref)	199/3				
Age 36–45 (years)	290/4	0.82	0.18	4.22	0.80
Age 46–55 (years)	475/26	2.89	0.10	12.3	0.09
Age 56–65 (years)	578/83	7.14	2.60	29.5	0.001
Age 66–75 (years)	446/113	11.9	4.35	49.2	<0.0001
Age 76–85 (years)	478/196	17.4	6.37	71.7	<0.0001
Age >85 (years)	124/104	44.8	15.9	187	<0.0001
Sex female (ref)	1,059/171				
Sex male	1,602/331	1.62	1.30	2.04	<0.0001
BMI 18.5–24.9 (kg·m^−2^) (ref)	873/167				
BMI 25–29.9 (kg·m^−2^)	977/178	0.99	0.78	1.29	0.99
BMI 30–34.9 (kg·m^−2^)	534/94	1.04	0.76	1.40	0.81
BMI ≥35 (kg·m^−2^)	277/63	1.77	1.22	2.56	0.003
No diabetes (ref)	2,119/333				
Diabetes	542/169	1.44	1.09	1.89	0.009
HbA1c <6.4% (ref)	48/6				
HbA1c 6.4–8 %	118/27	2.04	0.82	5.88	0.15
HbA1c 8.1–10%	61/14	2.65	0.95	8.16	0.07
HbA1c >10%	30/12	6.37	2.13	20.8	0.001
HbA1c not available	2,404/443	3.96	1.73	10.8	0.003
No hypertension (ref)	1,416/138				
Hypertension	1,245/364	1.27	0.99	1.61	0.056
No coronary artery disease (ref)	2,340/376				
Coronary artery disease	321/126	1.14	0.88	1.48	0.31
No chronic kidney disease (ref)	2,322/359				
Chronic kidney disease	339/143	1.42	1.10	1.82	0.007
No liver cirrhosis (ref)	2,643/493				
Liver cirrhosis	18/9	2.41	0.97	5.70	0.048

*OR, odds ratio; CI, confidence interval*.

### Risk of Mortality in Young/Middle-Aged, Older and Old Patients

To investigate the relationships of obesity and impaired metabolic health with the risk of mortality in different age groups, patients were divided into three age groups (Supplementary Table 4), with 1,068 young and middle-aged, 1,220 older age and 875 old age groups. Based on the similar sample sizes these three groups were equally strong powered for the investigation of the patient's characteristics with mortality in the statistical analyses. In multivariable regression analyses male sex was associated with a higher risk of mortality in the young/middle-aged and in the old age groups, but not in the older age group. BMI ≥35 kg·m^−2^ was associated with increased mortality in the young/middle-aged and in the older age groups, but not in the old age group. Diabetes was associated with increased mortality only in the old age group (Supplementary Table 5).

### Risk of Mortality in Subjects Stratified by Age and Obesity/Metabolic Health

To compare the contributions of advanced age vs. obesity and impaired metabolic health (diabetes and hypertension) to the mortality risk, we divided the patients into 12 subgroups based upon age and presence or absence of obesity, diabetes and hypertension. First, to investigate an additive effect of these parameters on the mortality risk, we divided the subjects in the three age groups based on the presence or absence of obesity, obesity + diabetes and obesity + diabetes + hypertension. Second, to investigate the impact of obesity + impaired metabolic health (diabetes and hypertension) on the risk of mortality more in detail, we compared the following 6 groups: (1) young and middle-aged without obesity, diabetes and hypertension (*N* = 593), (2) young and middle-aged with obesity, diabetes and hypertension (*N* = 31), (3) older age without obesity, diabetes and hypertension (*N* = 339), (4) older age with obesity, diabetes and hypertension (*N* = 148), (5) old age without obesity, diabetes and hypertension (*N* = 166) and (6) old age with obesity, diabetes and hypertension (*N* = 80).

**Figure 1:**
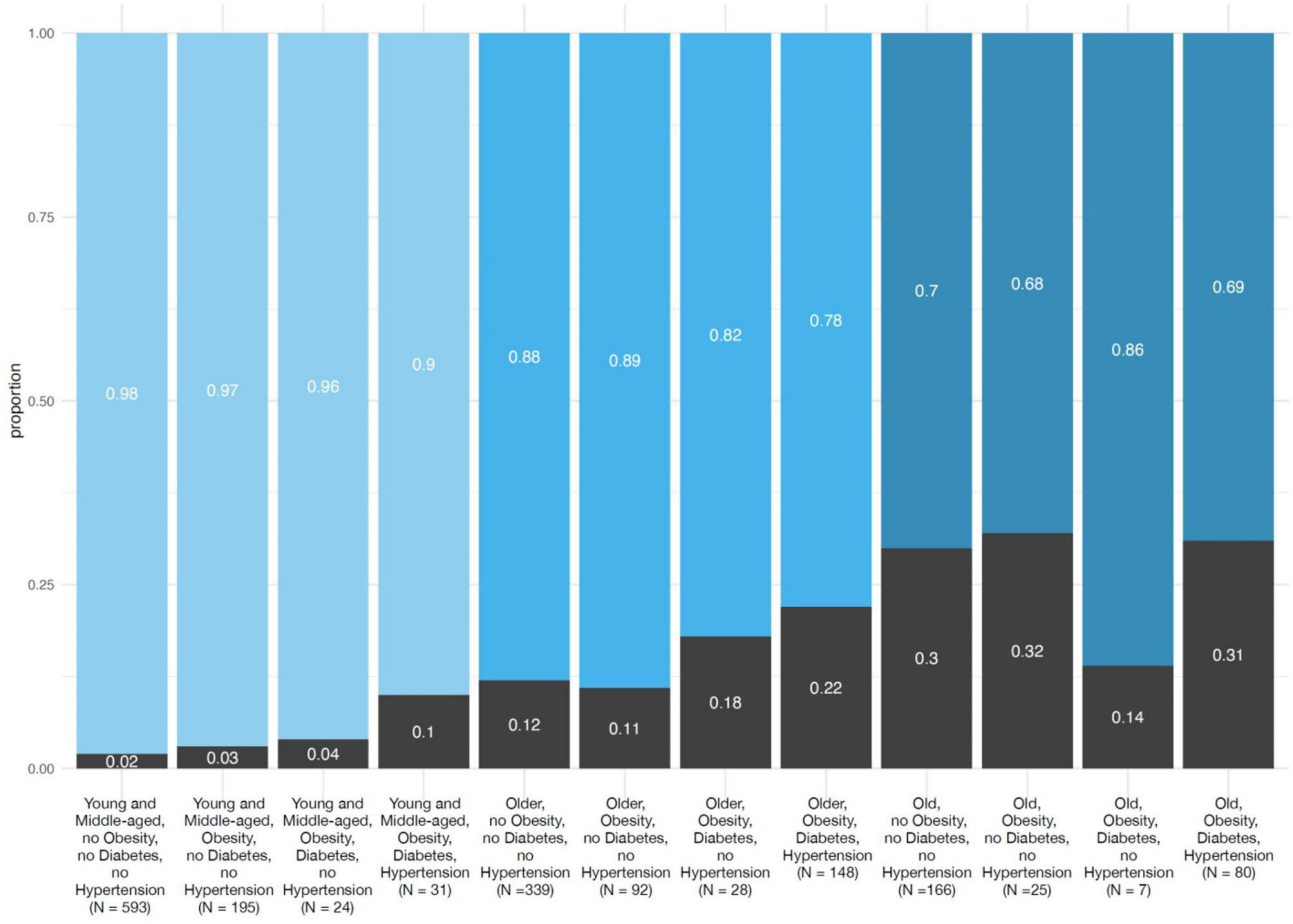
Proportion of COVID-19 patients who recovered and died divided in three age groups based on the presence or absence of obesity, diabetes and hypertension. All COVID-19 patients who recovered and died (*n* = 3,163) were first divided in three age groups (young and middle aged, 18–55 years, *n* = 1,068; older age, 56–75 years; *n* = 1,220 and old age, >75 years; *n* = 875) and subsequently divided in four groups based on the presence or absence of obesity (BMI ≥ 30 kg·m^−2^), and impaired metabolic health (diabetes and hypertension).

**Figure 2:**
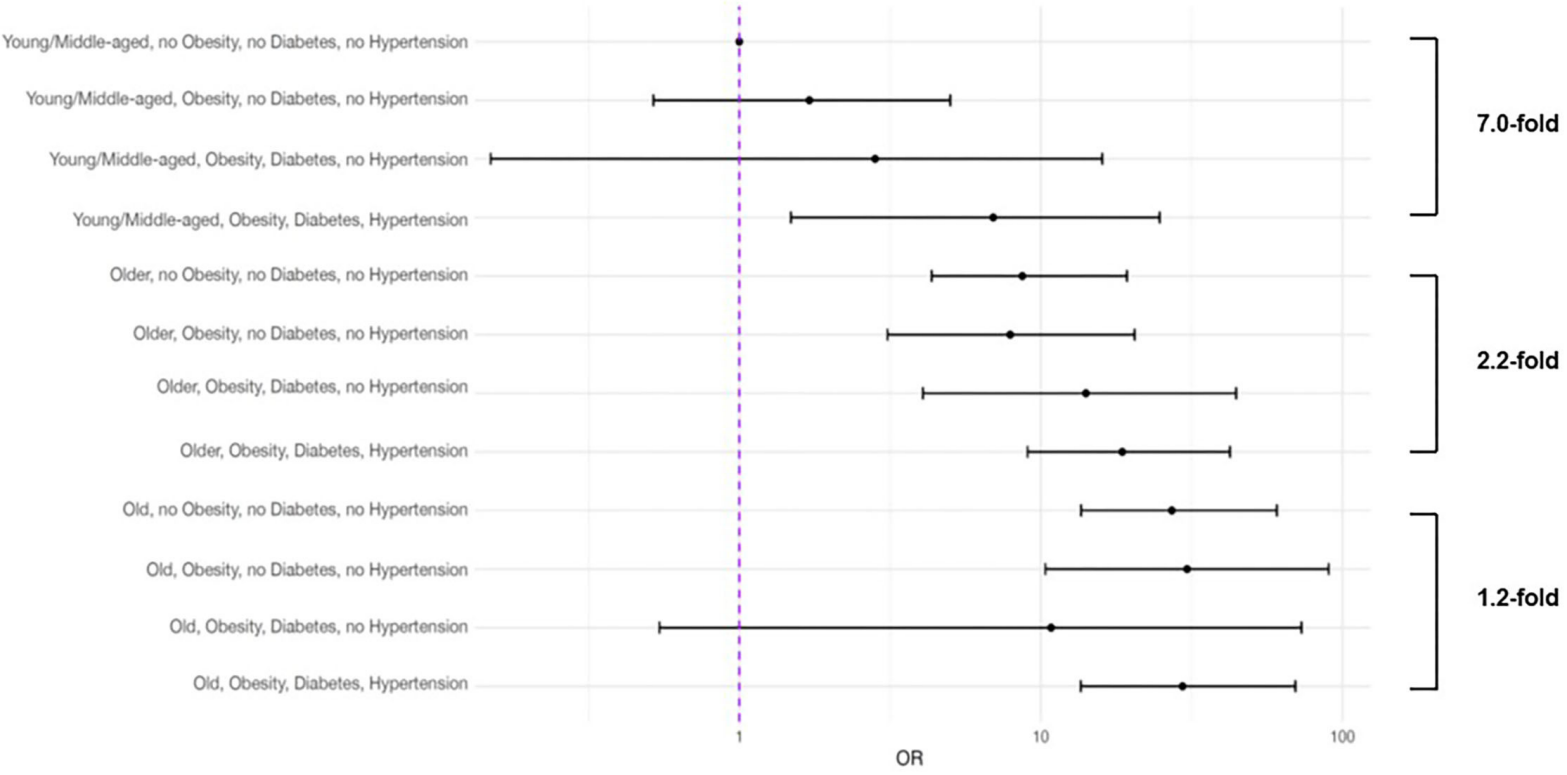
Multivariable relationships of selected anthropometrics, comorbidities and laboratory parameters with COVID-19-related mortality in three age groups based on the presence (unhealthy) or absence (healthy) of obesity, diabetes and hypertension. All COVID-19 patients who recovered and died (*n* = 3,163) were first divided in three age groups (young and middle aged, 18–55 years, *n* = 1,068; older age, 56–75 years; *n* = 1,220 and old age, >75 years; *n* = 875) and subsequently divided in two groups (*n* = 1,357) based on the presence (unhealthy) or absence (healthy) of obesity (BMI ≥ 30 kg·m^−2^), and impaired metabolic health (diabetes and hypertension). All parameters shown were included in the multivariable regression analysis.

When the age groups were stratified by the presence or absence of obesity and impaired metabolic health, both, older age and the presence of obesity and impaired metabolic health associated with increased risk of mortality (Figure 1). In the multivariable statistical model (Table 2, Model 1) moderately higher adjusted risks of mortality were observed in the young and middle-aged patients with obesity [*N* = 195; OR 1.75 (95% CI 0.53–5.13)] and obesity + diabetes [*N* = 24; OR 2.96 (95% CI 0.16–17.3)], which were statistically not significant, when compared to the young and middle-aged patients without obesity, diabetes or hypertension. However, when compared to the latter group, the adjusted risk of mortality was strongly increased in the young and middle-aged patients with obesity+impaired metabolic health [diabetes + hypertension; *N* = 31; OR 6.95 (95% CI 1.45–25.6)]. This group had a nearly 7-fold higher risk of mortality, compared to the young and middle-aged patients without obesity, diabetes or hypertension (Table 2, Model 1 and Figure 2).

**Table 2:** Multivariable relationships of three age groups based on the presence (unhealthy) or absence (healthy) of obesity, diabetes and hypertension and selected anthropometrics, comorbidities and laboratory parameters with COVID-19-related mortality.

**Characteristics**	**Model 1**				**Model 2**			
	**OR**	**Lower 95%CI**	**Upper 95%CI**	***p***	**OR**	**Lower 95%CI**	**Upper 95%CI**	***p***
Young/middle-aged–no obesity, no diabetes, no hypertension (ref.) (*N* = 593)								
Young/middle-age–obesity, no diabetes, no hypertension (*N* = 195)	1.75	0.53	5.13	0.32	1.55	0.47	4.60	0.45
Young/middle-aged–obesity, diabetes, no hypertension (*N* = 24)	2.96	0.16	17.3	0.32	2.81	0.14	17.1	0.35
Young/middle-aged–obesity, diabetes, hypertension (*N* = 31)	6.95	1.45	25.6	0.006	5.99	1.23	23.0	0.014
Older–no obesity, no diabetes, no hypertension (*N* = 339)	8.24	4.12	18.4	<0.0001	6.88	3.40	155	<0.0001
Older–obesity, no diabetes, no hypertension (*N* = 92)	7.70	3.01	20.0	<0.0001	5.88	2.25	15.5	0.0003
Older–obesity, diabetes, no hypertension (*N* = 28)	13.4	3.61	44.9	<0.0001	13.6	3.53	48.2	0.0001
Older–obesity, diabetes, hypertension (*N* = 148)	18.0	8.16	43.0	<0.0001	14.7	6.55	35.9	<0.0001
Old–no obesity, no diabetes, no hypertension (*N* = 166)	24.4	12.1	54.9	<0.0001	21.6	10.5	49.5	<0.0001
Old–obesity, no diabetes, no hypertension (*N* = 25)	29.6	9.88	88.8	<0.0000	24.6	7.94	75.6	<0.0001
Old–obesity, diabetes, no hypertension (*N* = 7)	7.47	0.37	52.4	0.08	6.62	0.32	48.5	0.10
Old–obesity, diabetes, hypertension (*N* = 80)	28.4	12.1	71.5	<0.0001	27.1	11.3	69.6	<0.0001
Sex male	1.38	0.98	1.95	0.07	1.28	0.90	1.83	0.18
HbA1c 6.4–8%	1.40	0.38	6.78	0.64	1.45	0.38	7.23	0.61
HbA1c 8.1–10%	1.99	0.50	10.1	0.36	2.78	0.67	14.6	0.19
HbA1c >10%	3.47	0.67	20.7	0.14	2.98	0.55	18.8	0.22
HbA1c unknown	2.34	0.72	10.6	0.20	2.48	0.74	11.5	0.18
Coronary artery disease	1.13	0.70	1.78	0.61	1.08	0.66	1.74	0.74
Chronic kidney disease	1.75	1.14	2.66	0.009	1.76	1.13	2.73	0.012
Liver cirrhosis	1.55	0.32	5.63	0.53	2.76	0.54	10.7	0.17
CRP 3–29 mg/L	-	-	-	-	1.77	0.58	7.71	0.37
CRP 30–69 mg/L	-	-	-	-	4.95	1.66	21.4	0.011
CRP 70–119 mg/L	-	-	-	-	5.32	1.74	23.3	0.009
CRP 120–179 mg/L	-	-	-	-	6.54	2.05	29.2	0.004
CRP 180–249 mg/L	-	-	-	-	17.4	5.01	81.8	<0.0001
CRP >249 mg/L	-	-	-	-	23.4	6.43	113	<0.0001
CRP unknown	-	-	-	-	6.56	2.31	27.6	0.002

*OR, odds ratio; CI, confidence interval; Model 1, adjusted for sex, HbA1c, coronary artery disease, chronic kidney disease and liver cirrhosis; Model 2, adjusted for sex, HbA1c, coronary artery disease, chronic kidney disease, liver cirrhosis and CRP*.

Older patients without obesity, diabetes or hypertension had a higher adjusted risk of mortality [*N* = 339; OR 8.24 (95% CI 4.12–18.4)], compared to young and middle-aged patients without obesity, diabetes or hypertension. This risk increased in the presence of obesity, diabetes and hypertension and older patients having all three risk factors (*N* = 148) had an adjusted OR for mortality of 18.0 (95% CI 8.16–43.0), compared to young and middle-aged patients without obesity, diabetes or hypertension. Interestingly, this risk was merely 2.2-fold higher than the risk of older patients without obesity, diabetes and hypertension (Table 2, Model 1 and Figure 2).

Old patients without obesity, diabetes and hypertension had a very high adjusted risk of mortality [*N* = 166; OR 24.4 (95% CI 12.1–54.9)], compared to young and middle-aged patients without obesity, diabetes or hypertension. However, in the old patients, obesity, diabetes or hypertension only weakly increased this risk [1.2-fold higher; *N* = 80; OR 28.4 (95% CI 12.1–71.5)] (Table 2, Model 1 and Figure 2).

Similar relationships were observed when patients were stratified in those with an uncomplicated and a severe (complicated phase and critical phase) course of the disease. For example, when compared to the young and middle-aged patients without obesity, diabetes or hypertension, the adjusted risk of severe COVID-19 was increased in the young and middle-aged patients with obesity + impaired metabolic health [diabetes + hypertension; *N* = 31; OR 2.60 (95% CI 1.87–3.64)]. Furthermore, this risk was comparable to the risk observed in older non-obese and metabolically healthy patients [*n* = 339; OR 2.66 (95% CI 2.01–3.52)] (Supplementary Table 6).

**Figure 3:**
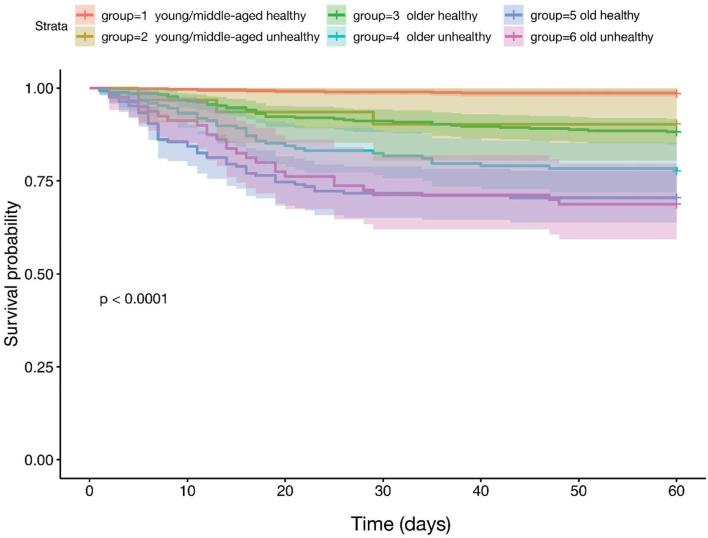
Kaplan-Meier survival comparing three age groups based on the presence (unhealthy) or absence (healthy) of obesity, diabetes and hypertension. All COVID-19 patients who recovered and died (*n* = 3,163) were first divided in three age groups (young and middle aged, 18–55 years, *n* = 1,068; older age, 56–75 years; *n* = 1,220 and old age, >75 years; *n* = 875) and subsequently divided in two groups (*n* = 1,357) based on the presence (unhealthy) or absence (healthy) of obesity (BMI ≥ 30 kg·m^−2^), and impaired metabolic health (diabetes and hypertension).

Among the patients who died, most deaths occurred within the first 2 weeks of follow-up. In Kaplan-Meier survival analyses young and middle-aged patients with obesity and impaired metabolic (diabetes + hypertension) health had a similar time-to-death to those in the older age group without obesity and impaired metabolic health (Figure 3). Compared to young and middle-aged patients without obesity and impaired metabolic health (group 1), the adjusted OR of mortality was 6.95 (95% CI 1.45–25.6) in the young and middle-aged group with obesity and impaired metabolic health (group 2), which was not statistically different from the risk in the older age group without obesity and impaired metabolic health [OR 8.24 (95% CI 4.12–18.4)] (Table 2, Model 1 and Figure 2).

We then explored parameters that may explain the elevated risk of COVID-19-related mortality with age, specifically in the absence of obesity and impaired metabolic health. We additionally adjusted our multivariable regression model for CRP levels (Table 2, Model 2). This resulted in an attenuation of the elevated risk of mortality observed in older and old patients without obesity and impaired metabolic health, when compared to young and middle-aged patients without obesity and impaired metabolic health, by 17 and 11%, respectively.

To address, whether the increased risk of mortality that associated with obesity and impaired metabolic health and that was very high, particularly in the group of young and middle-aged patients, may be predominantly driven by the risk in middle-aged patients, we also divided the patients in a younger (age 18–35 years) group and a middle-aged (age 36–55 years) group. Although the sample size was very low, only allowing an exploratory evaluation, we found that young patients with obesity and impaired metabolic health had a 4.2-fold higher risk of mortality, compared to the young patients without obesity and impaired metabolic health. A similarly increased risk (3.5-fold) was observed in the middle-aged patients with obesity and impaired metabolic health, compared to the middle-aged patients without obesity and impaired metabolic health (Supplementary Table 7).

## Discussion

Both, high BMI and adverse cardiometabolic status, are now established risk factors for severe COVID-19 (25). However, the risk attributed to these factors is considered to be lower than that of advanced age and perhaps also male sex. Nevertheless, the relative importance of these risk factors has not been well-studied. This knowledge gap may have direct public health implications, as metabolic risk factors–unlike age and sex–are modifiable (15–17). In this multi-national study, mostly including hospitalized patients with COVID-19, we found similar relationships of metabolic risk factors and adiposity, with COVID-19-related mortality, as were reported by previous studies (2–14). This allowed us to address an important question: to what extent does obesity, diabetes and hypertension, which were recently found to account for almost 60% of the COVID-19 hospitalizations in the United States (26), increase the risk of COVID-19-related mortality in younger patients, when compared to older patients. We found that an additive effect of obesity, diabetes and hypertension on the risk of COVID-19-related mortality exists. Compared to the respective older and old groups without these risk factors, the adjusted risk of mortality increased particularly strong in the young and middle-aged groups with these risk factors. In this respect, compared to young and middle-aged patients without obesity, patients merely having obesity only had a moderately increased adjusted mortality risk. This risk increased considerably in young and middle-aged patients with obesity and diabetes. Such an increase in risk was not observed in the older and old patients. Importantly, the presence of all three risk factors, obesity diabetes and hypertension, independently of other comorbidities and of sex, increased the risk of COVID-19-related mortality in younger and middle-aged patients to the risk level that we observed in older patients without these diseases. This finding is potentially of major public health relevance, as younger age is considered to protect from severe COVID-19.

Studies including COVID-19 patients from the United Kingdom reported that diabetes most strongly increased the risk of COVID-19-related mortality in younger patients (27, 28). Furthermore, data from the US CDC and the US Premier Healthcare Database of hospital-based patients with COVID-19 previously suggested that younger patients with obesity, diabetes or other comorbidities, have an increased risk of COVID-19-related death, that amounted to the risk often observed in older patients (21, 22). However, in those studies no adjustment for sex and comorbidities was done. In our study, diabetes was associated with an increased risk of COVID-19-related mortality in younger and in middle-aged patients, but this relationship was attenuated with adjustment for sex, BMI and other comorbidities. Thus, our findings indicate that obesity, diabetes and hypertension comprise a phenotype strongly associated with increased risk of COVID-19-related mortality in young and middle-aged patients, independently of other important determinants of severe COVID-19.

These findings may have several clinical implications. First, they support the recommendations of international medical societies, that obesity, diabetes and hypertension are important risk factors that should be critically considered by health care providers, when COVID-19 is being diagnosed in a patient. Intense clinical surveillance of these patients, particularly during the early stages of the disease, should be ensured. This approach is also supported by our findings of an increased mortality of obese and metabolically unhealthy COVID-19 patients during the first 2 weeks after diagnosis, independently of age.

Second, in view of the changing demographics of hospitalizations–with a substantial increase among patients <55 years relative to older people (21)–health care providers should not assume that younger individuals generally are at lower risk for severity of COVID-19. Consequently, younger people with these common risk factors should also be prioritized in vaccination strategies.

Third, there is increasing concern that SARS-CoV-2 will not only become an endemic virus and that an emergent coronavirus may cause severe disease in children (29–31), but that new variants of SARS-CoV-2 may evade the body's immune response, both in vaccinated and in not yet vaccinated people (29–35). Particularly the second year of the COVID-19 pandemic has been dominated by variants of concern (36, 37). Among them, mutations of the SARS-CoV-2 spike protein, the primary antigen, may be problematic, as most recently suggested for the Omicron (B.1.1.529) SARS-CoV-2 variant of concern^1^. In this respect obesity and diabetes may become even more important risk factors than currently considered. Obesity and impaired metabolic health may adversely influence the efficacy of SARS-CoV-2 vaccines (38, 39). In this respect, most recently some preliminary data indicate that obesity, diabetes and CVD may predispose for vaccine breakthrough COVID-19 infections (40–42). Premature immunesenescence, accelerated aging of the immune system, particularly of the CD4+ and CD8+ T cell compartments, has been found in people with obesity or type 2 diabetes (43–45). Intriguingly, as a mechanism explaining this observation, intact insulin signaling was observed to play an important role in modulating the body's immune response. Insulin receptor signaling has an impact on T cell glucose metabolism and amino acid handling. In rodents, insulin receptor-deficient T cells were found to have reduced inflammatory potential and poor protective immunity against H1N1 influenza infection (46). Considering that obesity, especially central adiposity, and impaired metabolic health, strongly associate with insulin resistance (47–49), and a healthy diet and exercise (50), as well as new dietary concepts to improve the gut microbiome (51) are very helpful to improve metabolic health, reduction of fat mass and a healthy diet may be critical for the coming months of the SARS-CoV-2 pandemic.

Fourth, most recently it was shown that, beyond the acute illness, substantial burden of health loss, including disorders of lipid metabolism, diabetes and obesity, is observed in COVID-19 survivors (52, 53). Although, this has not been investigated, yet, the presence of obesity and impaired metabolic health prior to the SARS-CoV-2 infection may particularly increase the burden of health loss in COVID-19 survivors. This may be problematic especially for younger patients, who may, thereby, experience a larger amount of years of life lost, than older patients.

A strength of our study is that the multi-center LEOSS registry prospectively collects epidemiological and clinical data based on a pre-specified protocol. Furthermore, the hospitals have the capacity to also monitor patients with asymptomatic or mild SARS-CoV-2 infections. However, there are several limitations. This study analyzed factors associated with disease course at initial presentation, not treatment, and cannot assess causality. We cannot rule out the presence of confounding from socioeconomic status, health insurance issues and access to health services and country specific testing capacities, among other factors. Some of these factors could be correlated with delayed diagnosis and therefore a more complicated clinical stage at initial presentation. Furthermore, the highest documentation rates were performed by University hospitals in larger cities; consequently, rural areas might be underrepresented. Finally, the sample size in the younger age groups was relatively small, most probably resulting from the fact that younger people generally are less often hospitalized with COVID-19 compared to middle-aged and older people. The small sample size in some of the groups may result in that a statistical error may occur from skewed group comparisons.

In conclusion, we found that obesity, diabetes and hypertension have an additive effect on COVID-19-related mortality and that this effect is particularly strong in young and middle-aged patients. Furthermore, we found that obesity, diabetes and hypertension increased the risk of COVID-19-related mortality in young and middle-aged patients to the risk level that we observed in older but metabolically healthy patients. Importantly, this increased risk was independent of other comorbidities and of sex. Awareness of health care providers about this strong impact of obesity and impaired metabolic health on the risk of COVID-19-related mortality may be critical to intensify surveillance of younger patients infected with SARS-CoV-2 and to motivate subjects at risk to lose weight and improve their metabolic health.

## Data Availability Statement

Patient data from the LEOSS registry are subject to the LEOSS governance, data use, and access policy (policy text available on https://leoss.net). Further inquiries can be directed to the corresponding author.

## Ethics Statement

The studies involving human participants were reviewed and approved by Local Ethics Committees of all participating centers and registered at the German Clinical Trails Register (DRKS, No. S00021145). Written informed consent for participation was not required for this study in accordance with the national legislation and the institutional requirements.

## Author Contributions

NS and KS analyzed the data and wrote the manuscript. NS, KS, MHe, and AB designed the study. CJ, AW, YK, SB, MMR, FH, MHa, CP, MHo, JD, KW, CR, JV, MSt, and BJ collected the data and contributed to the discussion. MHe, AF, RW, HP, MSo, MRo, AS, BG, MA, DL, MSc, and AB critically reviewed the manuscript and contributed to the discussion. NS is the guarantor of this study. All authors contributed to the article and approved the submitted version.

## Funding

The LEOSS registry was supported by the German Centre for Infection Research (DZIF) and the Willy Robert Pitzer Foundation. This article was also supported by funding from the German Centre for Diabetes Research (DZD).

## Leoss Study Group

We express our deep gratitude to all study teams supporting the LEOSS study. The LEOSS study group contributed at least 5 per mille to the analyses of this study: University Hospital Duesseldorf (Bjoern-Erik Ole Jensen), Technical University of Munich (Christoph Spinner), University Hospital Frankfurt (Maria Vehreschild), Hospital Ingolstadt (Stefan Borgmann), University Hospital Regensburg (Frank Hanses), University Hospital Jena (Maria Madeleine Ruethrich), Hospital Passau (Julia Lanznaster), Hospital Bremen-Center (Christiane Piepel), Hospital Dortmund (Martin Hower), Maria Hilf Hospital Moenchengladbach (Juergen vom Dahl), Johannes Wesling Hospital Minden Ruhr University Bochum (Kai Wille), University Hospital Augsburg (Christoph Römmele), University Hospital Freiburg (Siegbert Rieg), University Hospital Wuerzburg (Nora Isberner), Elisabeth Hospital Essen (Ingo Voigt), Marien Hospital Herne Ruhr University Bochum (Timm Westhoff), Municipal Hospital Karlsruhe (Christian Degenhardt), University Hospital Schleswig-Holstein Luebeck (Nadja Kaeding), Hospital Ernst von Bergmann (Lukas Tometten), Hospital Leverkusen (Lukas Eberwein), University Hospital Essen (Sebastian Dolff), Elbland Hospital Riesa (Joerg Schubert), University Hospital of Giessen and Marburg (Janina Trauth), University Hospital Ulm (Beate Gruener), Robert-Bosch-Hospital Stuttgart (Katja Rothfuss), Nephrological Center Villingen-Schwenningen (Bernd Hohenstein), University Hospital Tuebingen (Silvio Nadalin), Bundeswehr Hospital Koblenz (Dominic Rauschning), University Hospital Erlangen (Richard Strauss), Helios Hospital Pirna (Christian Riedel), University Hospital Saarland (Robert Bals), University Hospital Cologne (Norma Jung), Hacettepe University (Murat Akova), Hospital Braunschweig (Jan Kielstein), Hospital of the Augustinian Cologne (Stefani Roeseler), Catholic Hospital Bochum (St. Josef Hospital), Ruhr University Bochum (Kerstin Hellwig), Tropical Clinic Paul-Lechler Hospital Tuebingen (Claudia Raichle), Hospital Preetz (Helga Peetz), Hospital St. Joseph-Stift Dresden (Lorenz Walter), Malteser Hospital St. Franziskus Flensburg (Milena Milovanovic), Medical School Hannover (Gernot Beutel), National MS Center Melsbroek (Marie D'Hooghe), Practice at Ebertplatz Cologne (Christoph Wyen), University Hospital Dresden (Katja de With), University Hospital Schleswig-Holstein - Kiel (Anette Friedrichs).

The LEOSS study infrastructure group: Jörg Janne Vehreschild (Goethe University Frankfurt), Carolin E. M. Jakob (University Hospital of Cologne), Lisa Pilgram (Goethe University Frankfurt), Melanie Stecher (University Hospital of Cologne), Max Schons (University Hospital of Cologne), Susana Nunes de Miranda (University Hospital of Cologne), Clara Bruenn (University Hospital of Cologne), Nick Schulze (University Hospital of Cologne), Sandra Fuhrmann (University Hospital of Cologne), Annika Claßen (University Hospital of Cologne), Bernd Franke (University Hospital of Cologne), Fabian Praßer (Charité, Universitätsmedizin Berlin) und Martin Lablans (University Medical Center Mannheim).

## Conflict of Interest

The authors declare that the research was conducted in the absence of any commercial or financial relationships that could be construed as a potential conflict of interest.

## Publisher's Note

All claims expressed in this article are solely those of the authors and do not necessarily represent those of their affiliated organizations, or those of the publisher, the editors and the reviewers. Any product that may be evaluated in this article, or claim that may be made by its manufacturer, is not guaranteed or endorsed by the publisher.

## Footnotes

^1^ https://www.who.int/news/item/26-11-2021-classification-of-omicron-(b.1.1.529)-sars-cov-2-variant-of-concern
