# Supplementary material for: Obesity and Impaired Metabolic Health Increase Risk of COVID-19-Related Mortality in Young and Middle-Aged Adults to the Level Observed in Older People: The LEOSS Registry
Source: Front Med (Lausanne). 2022 May 11;9:875430. doi: 10.3389/fmed.2022.875430 (PMC9131026; doi:10.3389/fmed.2022.875430)
Supplement: Supplementary file 1 [file Data_Sheet_1.docx]

**Supplementary information**

**Obesity and Impaired Metabolic Health Increase Risk of COVID-19-Related Mortality in Young and Middle-Aged Adults to the Level Observed in Older People: the LEOSS Registry**

Norbert Stefan, MD^1,2,3*^ Katrin Sippel, PhD^1,2,3*^ Martin Heni,PhD^1,2,3^ Andreas Fritsche, PhD^1,2,3^ Robert Wagner, PhD^1,2,3^ Carolin E. M. Jakob,^4,5^ Hubert Preißl, PhD^1,3^ Alexander von Werder, MD^5,6^ Yascha Khodamoradi, MD^7^ Stefan Borgmann, MD^8^ Maria Madeleine Rüthrich, MD^9^ Frank Hanses, MD^10^ Martina Haselberger, MD^11^ Christiane Piepel, MD^12^ Martin Hower, MD^13^ Juergen vom Dahl, MD^14^ Kai Wille, MD^15^ Christoph Römmele, MD^16^ Janne Vehreschild, MD^3,4,17^ Melanie Stecher,^4,5^ Michele Solimena, PhD^3,18^ Michael Roden, MD^3,19,20^ Annette Schürmann, PhD^3,21^ Baptist Gallwitz, MD^2^ Martin Hrabe de Angelis, PhD^3,22^ David S. Ludwig, MD^23^ Matthias B. Schulze, DrPH^3,24^ Bjoern Erik Ole Jensen, MD^25#^ Andreas L. Birkenfeld, MD^1,2,3#^ on behalf of the LEOSS Study Group

**Suppl. Table 1.** Characteristics of the patients with COVID-19

| **Characteristics** | **Category** | **Recovered** | **Died** |
| --- | --- | --- | --- |
|  |  | 2661 (84.13 %) | 502 (15.87 %) |
| **Age** |  |  |  |
|  | Age 18-25 | 71 (100 %) | 0 (0 %) |
|  | Age 26-35 | 199 (98.51 %) | 3 (1.49 %) |
|  | Age 36-45 | 290 (98.64 %) | 4 (1.36 %) |
|  | Age 46-55 | 475 (94.81 %) | 26 (5.19 %) |
|  | Age 56-65 | 578 (87.44 %) | 83 (12.56 %) |
|  | Age 65-75 | 446 (79.79 %) | 113 (20.21 %) |
|  | Age 76-85 | 478 (73.88 %) | 169 (26.12 %) |
|  | Age >85 | 124 (54.39 %) | 104 (45.61 %) |
| **Sex** |  |  |  |
|  | Male | 1602 (82.88 %) | 331 (17.12 %) |
|  | Female | 1059 (86.1 %) | 171 (13.9 %) |
| **BMI** |  |  |  |
|  | 18.5 -24.9 | 873 (83.94 %) | 167 (16.06 %) |
|  | 25 - 29.9 | 977 (84.59 %) | 178 (15.41 %) |
|  | 30 - 34.9 | 534 (85.03 %) | 94 (14.97 %) |
|  | >= 35 | 277 (81.47 %) | 63 (18.53 %) |
| **Diabetes Status** |  |  |  |
|  | No diabetes | 2119 (86.42 %) | 333 (13.58 %) |
|  | Diabetes | 542 (76.23 %) | 169 (23.77 %) |
| **Diabetes Type** |  |  |  |
|  | Diabetes type 1 | 17 (89.47 %) | 2 (10.53 %) |
|  | Diabetes type 2 | 497 (76.46 %) | 153 (23.54 %) |
| **Diabetes Duration** |  |  |  |
|  | < 1 year | 15 (88.24 %) | 2 (11.76 %) |
|  | 1 -10 years | 67 (75.28 %) | 22 (24.72 %) |
|  | > 10 years | 73 (71.57 %) | 29 (28.43 %) |
|  | unknown | 2506 (84.81 %) | 449 (15.19 %) |
| **Diabetes Treatment** |  |  |  |
|  | Insulin | 174 (76.32 %) | 54 (23.68 %) |
|  | Metformin | 204 (83.27 %) | 41 (16.73 %) |
|  | Sulfonylureas | 17 (89.47 %) | 2 (10.53 %) |
|  | GLP-1-Analogs | 20 (83.33 %) | 4 (16.67 %) |
|  | DPP4-Inhibitors | 53 (79.1 %) | 14 (20.9 %) |
|  | SGLT2-Inhibitors | 34 (82.93 %) | 7 (17.07 %) |
| **HbA1c** |  |  |  |
|  | <6.4 | 48 (88.89 %) | 6 (11.11 %) |
|  | 6.4 - 8 % | 118 (81.38 %) | 27 (18.62 %) |
|  | 8.1 - 10% | 61 (81.33 %) | 14 (18.67 %) |
|  | >10% | 30 (71.43 %) | 12 (28.57 %) |
|  | not available | 2404 (84.44 %) | 443 (15.56 %) |
| **Hypertension** |  |  |  |
|  | yes | 1245 (77.38 %) | 364 (22.62 %) |
|  | no | 1416 (91.12 %) | 138 (8.88 %) |
|  |  |  |  |
| **Coronary Artery Disease** |  |  |  |
|  | yes | 321 (71.81 %) | 126 (28.19 %) |
|  | no | 2340 (86.16 %) | 376 (13.84 %) |
| **Chronic Kidney Disease** |  |  |  |
|  | yes | 339 (70.33 %) | 143 (29.67 %) |
|  | no | 2322 (86.61 %) | 359 (13.39 % |
| **Chronic Liver Disease** |  |  |  |
|  | yes | 55 (74.32 %) | 19 (25.68 %) |
|  | no | 2606 (84.36 %) | 483 (15.64 %) |
| **Liver Cirrhosis** |  |  |  |
|  | yes | 18 (66.67 %) | 9 (33.33 %) |
|  | no | 2643 (84.28 %) | 493 (15.72 %) |
| **CRP** |  |  |  |
|  | < 3 mg/L | 286 (96.95 %) | 9 (3.05 %) |
|  | 3- 29 mg/L | 732 (91.04 %) | 72 (8.96 %) |
|  | 30 - 69 mg/L | 343 (82.25 %) | 74 (17.75 %) |
|  | 70 - 119 mg/L | 263 (77.13 %) | 78 (22.87 %) |
|  | 120 - 179 mg/L | 160 (72.73 %) | 60 (27.27 %) |
|  | 180 - 249 mg/L | 68 (64.76 %) | 37 (35.24 %) |
|  | > 249 mg/L | 41 (53.25 %) | 36 (46.75 %) |
|  | not available | 768 (84.96 %) | 136 (15.04 %) |
| **IL6** |  |  |  |
|  | < 1.8 pg/mL | 25 (92.59 %) | 2 (7.41 %) |
|  | 1.8 - 49 pg/mL | 430 (95.56 %) | 20 (4.44 %) |
|  | 50 - 199 pg/mL | 184 (76.03 %) | 58 (23.97 %) |
|  | 200 - 499 pg/mL | 30 (49.18 %) | 31 (50.82 %) |
|  | 500 - 999 pg/mL | 5 (31.25 %) | 11 (68.75 %) |
|  | >1000 pg/mL | 8 (34.78 %) | 15 (65.22 %) |
|  | not available | 1979 (84.43 %) | 365 (15.57 %) |
| **Serum-Creatinine** |  |  |  |
|  | Normal | 1337 (90.4 %) | 142 (9.6 %) |
|  | >ULN | 357 (71.69 %) | 141 (28.31 %) |
|  | >2x ULN | 92 (63.01 %) | 54 (36.99 %) |
|  | >5x ULN | 29 (60.42 %) | 19 (39.58 %) |
|  | >10x ULN | 11 (100 %) | 0 (0 %) |
|  | not available | 835 (85.12 %) | 146 (14.88 %) |
| **AST** |  |  |  |
|  | Normal | 1002 (87.28 %) | 146 (12.72 % |
|  | >ULN | 442 (80.66 %) | 106 (19.34 %) |
|  | >2x ULN | 114 (76 %) | 36 (24 %) |
|  | >5x ULN | 13 (61.9 %) | 8 (38.1 %) |
|  | >10x ULN | 1 (14.29 %) | 6 (85.71 %) |
|  | >20x ULN | 2 (50 %) | 2 (50 %) |
|  | not available | 1087 (84.59 %) | 198 (15.41 %) |
| **ALT** |  |  |  |
|  | Normal | 1223 (84.05 %) | 232 (15.95 %) |
|  | >ULN | 358 (87.32 %) | 52 (12.68 %) |
|  | >2x ULN | 73 (80.22 %) | 18 (19.78 %) |
|  | >5x ULN | 12 (63.16 %) | 7 (36.84 %) |
|  | >10x ULN | 0 (%) | 0 (%) |
|  | >10x ULN | 0 (%) | 0 (%) |
|  | not available | 995 (83.75 %) | 193 (16.25 %) |
| **GGT** |  |  |  |
|  | Normal | 992 (87.02 %) | 148 (12.98 %) |
|  | >ULN | 334 (83.92 %) | 64 (16.08 %) |
|  | >2x ULN | 177 (78.32 %) | 49 (21.68 %) |
|  | >5x ULN | 40 (68.97 %) | 18 (31.03 %) |
|  | >10x ULN | 14 (42.42 %) | 19 (57.58 %) |
|  | >20x ULN | 0 (%) | 0 (%) |
|  | not available | 1104 (84.4 %) | 204 (15.6 %) |

**Suppl. Table 2.** Univariable relationships of anthropometrics, comorbidities and laboratory parameters with COVID-19-related mortality

| **Characteristics** | **OR** | **Lower 95% CI** | **Upper 95% CI** | **p** |
| --- | --- | --- | --- | --- |
| Age 18-25 years | 0.00 | 0.0000 | 0.0000 | 0.97 |
| Age 26-35 years (ref) |  |  |  |  |
| Age 36-45 years | 0.91 | 0.20 | 4.69 | 0.91 |
| Age 46-55 years | 3.63 | 1.26 | 15.35 | 0.036 |
| Age 56-65 years | 9.53 | 3.52 | 39.1 | 0.0001 |
| Age 66-75 years | 16.8 | 6.24 | 68.8 | <0.0001 |
| Age 76-85 years | 23.5 | 8.77 | 95.7 | <0.0001 |
| Age >85 years | 55.6 | 20.4 | 229 | <0.0001 |
| Sex female (ref) |  |  |  |  |
| Sex male | 1.28 | 1.05 | 1.57 | 0.016 |
| BMI 18.5 - 24.9 (kg∙m^-2^) (ref) |  |  |  |  |
| BMI 25 - 29.9 (kg∙m^-2^) | 0.95 | 0.76 | 1.20 | 0.68 |
| BMI 30 - 34.9 (kg∙m^-2^) | 0.92 | 0.70 | 1.21 | 0.55 |
| BMI ≥35 (kg∙m^-2^) | 1.19 | 0.86 | 1.63 | 0.29 |
| No diabetes (ref) |  |  |  |  |
| Diabetes | 1.98 | 1.61 | 2.44 | <0.0001 |
| No Hypertension (ref) |  |  |  |  |
| Hypertension | 3.00 | 2.44 | 3.71 | <0.0001 |
| No Coronary Artery Disease (ref) |  |  |  |  |
| Coronary Artery Disease | 2.44 | 1.93 | 3.08 | <0.0001 |
| No Chronic Kidney Disease (ref) |  |  |  |  |
| Chronic Kidney Disease | 2.73 | 2.18 | 3.41 | <0.0001 |
| No Chronic Liver Disease (ref) |  |  |  |  |
| Chronic Liver Disease | 1.86 | 1.07 | 3.11 | 0.021 |
| No Liver Cirrhosis (ref) |  |  |  |  |
| Liver Chirrhosis | 2.68 | 1.14 | 5.85 | 0.017 |
| No Insulin (ref) |  |  |  |  |
| Insulin | 1.78 | 1.28 | 2.44 | 0.0005 |
| Insulin unknown | 3.27 | 1.84 | 5.67 | <0.0001 |
| No Metformin (ref) |  |  |  |  |
| Metformin | 1.07 | 0.75 | 1.50 | 0.70 |
| No Sulfonylureas (ref) |  |  |  |  |
| Sulfonylureas | 0.62 | 0.10 | 2.18 | 0.53 |
| No GLP-1-Analogs(ref) |  |  |  |  |
| GLP-1-Analogs | 1.06 | 0.31 | 2.82 | 0.91 |
| No DPP4-Inhibitors (ref) |  |  |  |  |
| DPP4-Inhibitors | 1.41 | 0.75 | 2.49 | 0.26 |
| No SGLT2-Inhibitors (ref) |  |  |  |  |
| SGLT2-Inhibitors | 1.09 | 0.44 | 2.33 | 0.83 |
| HbA1c <6.4% (ref) |  |  |  |  |
| HbA1c 6.4% - 8% | 1.83 | 0.75 | 5.15 | 0.21 |
| HbA1c 8.1% - 10% | 1.84 | 0.68 | 5.51 | 0.25 |
| HbA1c >10% | 3.20 | 1.12 | 10.0 | 0.035 |
| HbA1c not available | 1.47 | 0.68 | 3.86 | 0.37 |
| CRP <3 mg/L (ref) |  |  |  |  |
| CRP 3 - 29 mg/L | 3.13 | 1.63 | 6.79 | 0.002 |
| CRP 30 - 69 mg/L | 6.86 | 3.55 | 14.9 | <0.0001 |
| CRP 70 - 119 mg/L | 9.42 | 4.88 | 20.5 | <0.0001 |
| CRP 120 - 179 mg/L | 11.9 | 6.05 | 26.3 | <0.0001 |
| CRP 180 - 249 mg/L | 17.3 | 8.30 | 39.7 | <0.0001 |
| CRP >249 mg/L | 27.9 | 13.0 | 65.6 | 0.0000 |
| CRP not available | 5.63 | 2.99 | 12.0 | <0.0001 |
| IL6 <1.8 pg/mL (ref) |  |  |  |  |
| IL6 1.8 - 49 pg/mL | 0.58 | 0.16 | 3.77 | 0.48 |
| IL6 50 - 199 pg/mL | 3.94 | 1.13 | 24.9 | 0.068 |
| IL6 200 - 499 pg/mL | 12.9 | 3.43 | 84.7 | 0.001 |
| IL6 500 - 999 pg/mL | 27.5 | 5.45 | 219 | 0.0003 |
| IL6 >1000 pg/mL | 23.4 | 5.22 | 171 | 0.0002 |
| IL6 not available | 2.31 | 0.68 | 14.4 | 0.26 |
| Creatinine Normal (ref) |  |  |  |  |
| Creatinine >ULN | 3.72 | 2.87 | 4.83 | <0.0001 |
| Creatinine >2x ULN | 5.53 | 3.77 | 8.05 | <0.0001 |
| Creatinine >5x ULN | 4.47 | 2.47 | 7.83 | <0.0001 |
| Creatinine not available | 1.65 | 1.29 | 2.11 | 0.0001 |
| AST Normal (ref) |  |  |  |  |
| AST >ULN | 1.65 | 1.25 | 2.16 | 0.0004 |
| AST >2x ULN | 2.17 | 1.42 | 3.25 | 0.0002 |
| AST >5x ULN | 4.22 | 1.65 | 10.20 | 0.002 |
| AST >10x ULN | 41.2 | 6.97 | 780 | 0.0006 |
| AST >20x ULN | 6.86 | 0.82 | 57.6 | 0.055 |
| AST not available | 1.25 | 0.99 | 1.58 | 0.058 |
| ALT Normal (ref) |  |  |  |  |
| ALT >ULN | 0.77 | 0.55 | 1.05 | 0.11 |
| ALT >2x ULN | 1.30 | 0.74 | 2.17 | 0.34 |
| ALT >5x ULN | 3.08 | 1.13 | 7.73 | 0.020 |
| ALT not available | 1.02 | 0.83 | 1.26 | 0.83 |
| GGT Normal (ref) |  |  |  |  |
| GGT >ULN | 1.28 | 0.93 | 1.76 | 0.12 |
| GGT >2x ULN | 1.86 | 1.28 | 2.65 | 0.0008 |
| GGT >5x ULN | 3.02 | 1.65 | 5.32 | 0.0002 |
| GGT >10x ULN | 9.10 | 4.49 | 18.9 | <0.0001 |
| GGT not available | 1.24 | 0.99 | 1.56 | 0.066 |
| Ketone Bodies Negative - (ref) |  |  |  |  |
| Ketone Bodies Positive + | 0.89 | 0.55 | 1.38 | 0.60 |
| Ketone Bodies Positive ++ | 0.45 | 0.15 | 1.04 | 0.09 |
| Ketone Bodies Positive +++ | 0.40 | 0.10 | 1.16 | 0.14 |
| Ketone Bodies not available | 0.74 | 0.59 | 0.92 | 0.007 |

OR, odds ratio; CI, confidence interval

**Suppl. Table 3.** Multivariable relationships of anthropometrics, comorbidities and laboratory parameters with COVID-19-related mortality

| **Characteristics OR Lower Upper**  **95% CI 95% CI p** | | | | |
| --- | --- | --- | --- | --- |
| Age 18-25 years | 0.00 | 0.00 | 0.00 | 0.96 |
| Age 36-45 years | 0.90 | 0.19 | 4.71 | 0.89 |
| Age 46-55 years | 2.38 | 0.79 | 10.3 | 0.17 |
| Age 56-65 years | 5.52 | 1.95 | 23.3 | 0.005 |
| Age 66-75 years | 8.99 | 3.17 | 37.9 | 0.0003 |
| Age 76-85 years | 14.8 | 5.23 | 62.4 | <0.0001 |
| Age >85 years | 41.2 | 14.1 | 176 | <0.0001 |
| Sex male | 1.61 | 1.26 | 2.07 | 0.0002 |
| BMI 25 - 29.9 (kg∙m^-2^) | 0.99 | 0.75 | 1.30 | 0.92 |
| BMI 30 - 34.9 (kg∙m^-2^) | 1.01 | 0.72 | 1.39 | 0.97 |
| BMI >= 35 (kg∙m^-2^) | 1.54 | 1.02 | 2.31 | 0.038 |
| Diabetes | 1.57 | 1.08 | 2.28 | 0.018 |
| Insulin | 0.96 | 0.61 | 1.50 | 0.85 |
| Insulin unknown | 1.48 | 0.71 | 3.04 | 0.29 |
| Metformin | 0.66 | 0.41 | 1.04 | 0.080 |
| Sulfonylureas | 0.36 | 0.05 | 1.45 | 0.21 |
| GLP-1 Analogs | 0.55 | 0.14 | 1.70 | 0.33 |
| DPP4-Inhibitors | 0.68 | 0.33 | 1.36 | 0.29 |
| SGLT2-Inhibitors | 1.04 | 0.37 | 2.63 | 0.93 |
| HbA1c 6.4% - 8% | 2.53 | 0.92 | 7.96 | 0.089 |
| HbA1c 8.1% - 10% | 3.72 | 1.19 | 12.7 | 0.028 |
| HbA1c >10% | 6.23 | 1.79 | 23.3 | 0.005 |
| HbA1c not available | 4.36 | 1.73 | 12.9 | 0.004 |
| CRP 3- 29 mg/L | 2.10 | 1.01 | 4.92 | 0.064 |
| CRP 30 - 69 mg/L | 4.23 | 2.02 | 9.97 | 0.0003 |
| CRP 70 - 119 mg/L | 5.71 | 2.72 | 13.5 | <0.0001 |
| CRP 120 - 179 mg/L | 6.56 | 3.01 | 15.9 | <0.00001 |
| CRP 180 - 249 mg/L | 7.50 | 3.19 | 19.3 | <0.00001 |
| CRP > 249 mg/L | 12.9 | 5.24 | 34.4 | <0.00001 |
| CRP not available | 5.13 | 2.06 | 14.0 | 0.0008 |
| IL6 1.8 - 49 pg/mL | 0.290 | 0.06 | 2.20 | 0.16 |
| IL6 50 - 199 pg/mL | 0.88 | 0.19 | 6.65 | 0.89 |
| IL6 200 - 499 pg/mL | 1.98 | 0.39 | 15.7 | 0.45 |
| IL6 500 - 999 pg/mL | 6.11 | 0.89 | 61.1 | 0.086 |
| IL6 >1000 pg/mL | 5.92 | 0.96 | 53.4 | 0.075 |
| IL6 not available | 0.81 | 0.18 | 6.00 | 0.81 |
| Creatinine >ULN | 1.74 | 1.26 | 2.40 | 0.0007 |
| Creatinine >2x ULN | 1.68 | 1.03 | 2.71 | 0.035 |
| Creatinine >5x ULN | 1.69 | 0.78 | 3.60 | 0.17 |
| Creatinine not available | 1.28 | 0.67 | 2.37 | 0.44 |
| AST >ULN | 1.38 | 0.97 | 1.97 | 0.073 |
| AST >2x ULN | 1.54 | 0.80 | 2.94 | 0.20 |
| AST >5x ULN | 1.41 | 0.36 | 5.27 | 0.61 |
| AST >10x ULN | 13.0 | 0.83 | 552 | 0.12 |
| AST >20x ULN | 3.95 | 0.11 | 249 | 0.49 |
| AST not available | 0.86 | 0.51 | 1.43 | 0.56 |
| ALT >ULN | 0.67 | 0.42 | 1.03 | 0.071 |
| ALT >2x ULN | 0.98 | 0.42 | 2.24 | 0.97 |
| ALT >5x ULN | 1.44 | 0.23 | 7.69 | 0.69 |
| ALT not available | 1.34 | 0.76 | 2.37 | 0.31 |
| GGT >ULN | 1.10 | 0.74 | 1.61 | 0.64 |
| GGT >2x ULN | 1.11 | 0.69 | 1.77 | 0.67 |
| GGT >5x ULN | 1.25 | 0.60 | 2.52 | 0.53 |
| GGT >10x ULN | 4.36 | 1.62 | 12.1 | 0.004 |
| GGT not available | 0.93 | 0.57 | 1.48 | 0.76 |
| Ketone Bodies Positive + | 1.16 | 0.67 | 1.97 | 0.60 |
| Ketone Bodies Positive ++ | 0.59 | 0.18 | 1.60 | 0.34 |
| Ketone Bodies Positive +++ | 0.51 | 0.11 | 1.72 | 0.33 |
| Ketone Bodies not available | 0.89 | 0.66 | 1.18 | 0.40 |
| Hypertension | 1.22 | 0.94 | 1.59 | 0.13 |
| Coronary Artery Disease | 1.10 | 0.82 | 1.45 | 0.53 |
| Chronic Kidney Disease | 1.18 | 0.87 | 1.59 | 0.28 |
| Chronic Liver Disease | 1.78 | 0.92 | 3.30 | 0.074 |
| Liver Cirrhosis | 2.03 | 0.75 | 5.26 | 0.15 |

OR, odds ratio; CI, confidence interval

**Suppl. Table 4.** Characteristics of the patients with COVID-19 divided in three age groups

| **Characteristics** | **Recovered**  **(Young/Middle-aged)** | **Died**  **(Young/Middle-aged)** | **Recovered**  **(Older)** | **Died**  **(Older)** | **Recovered**  **(Old)** | **Died**  **(Old)** |
| --- | --- | --- | --- | --- | --- | --- |
| **Total** | 1035 (96.91 %) | 33 (3.09 %) | 1024 (83.93 %) | 196 (16.07 %) | 602 (68.8 %) | 273 (31.2 %) |
| **Age** |  |  |  |  |  |  |
| Age 18-25 (years) | 71 (100 %) | 0 (0 %) |  |  |  |  |
| Age 26-35 (years) | 199 (98.51 %) | 3 (1.49 %) |  |  |  |  |
| Age 36-45 (years) | 290 (98.64 % | 4 (1.36 %) |  |  |  |  |
| Age 46-55 (years) | 475 (94.81 %) | 26 (5.19 %) |  |  |  |  |
| Age 56-65 (years) |  |  | 578 (87.44 %) | 83 (12.56 %) |  |  |
| Age 66-75 (years) |  |  | 446 (79.79 %) | 113 (20.21 %) |  |  |
| Age 76-85 (years) |  |  |  |  | 478 (73.88 %) | 169 (26.12 %) |
| Age >85 (years) |  |  |  |  | 124 (54.39 %) | 104 (45.61 %) |
| **Sex** |  |  |  |  |  |  |
| Male | 645 (95.84 %) | 28 (4.16 %) | 655 (83.23 %) | 132 (16.77 %) | 302 (63.85 %) | 171 (36.15 %) |
| Female | 390 (98.73 %) | 5 (1.27 %) | 369 (85.22 %) | 64 (14.78 %) | 300 (74.63 %) | 102 (25.37 %) |
| BMI |  |  |  |  |  |  |
| 18.5 -24.9 (kg‧m^-2^) | 352 (98.6 %) | 5 (1.4 %) | 276 (85.98 %) | 45 (14.02 %) | 245 (67.68 %) | 117 (32.32 %) |
| 25 - 29.9 (kg‧m^-2^) | 369 (97.11 %) | 11 (2.89 %) | 390 (85.15 %) | 68 (14.85 %) | 218 (68.77 %) | 99 (31.23 %) |
| 30 - 34.9 (kg‧m^-2^) | 198 (96.12 %) | 8 (3.88 %) | 230 (85.82 %) | 38 (14.18 %) | 106 (68.83 %) | 48 (31.17 %) |
| ≥35 (kg‧m^-2^) | 116 (92.8 %) | 9 (7.2 %) | 128 (73.99 %) | 45 (26.01 %) | 33 (78.57 %) | 9 (21.43 %) |
| **Diabetes Status** |  |  |  |  |  |  |
| No Diabetes | 939 (97.51 %) | 24 (2.49 %) | 746 (85.75 %) | 124 (14.25 %) | 434 (70.11 %) | 185 (29.89 %) |
| Diabetes | 96 (91.43 %) | 9 (8.57 %) | 278 (79.43 %) | 72 (20.57 %) | 168 (65.62 %) | 88 (34.38 %) |
| **HbA1c** |  |  |  |  |  |  |
| <6.4 (%) | 6 (85.71 %) | 1 (14.29 %) | 21 (95.45 %) | 1 (4.55 %) | 21 (84 %) | 4 (16 %) |
| 6.4 – 8 (%) | 18 (90 %) | 2 (10 %) | 64 (85.33 %) | 11 (4.67 %) | 36 (72 %) | 14 (28 %) |
| 0.1 – 10 (%) | 11 (91.67 %) | 1 (8.33 %) | 36 (85.71 %) | 6 (14.29 %) | 14 (66.67 %) | 7 (33.33 %) |
| >10 (%) | 10 (90.91 %) | 1 (9.09 %) | 15 (62.5 %) | 9 (37.5 %) | 5 (71.43 %) | 2 (28.57 %) |
| unknown: | 990 (97.25 %) | 28 (2.75 %) | 888 (84.01 %) | 169 (15.99 %) | 526 (68.13 %) | 246 (31.87 %) |
| **Hypertension** |  |  |  |  |  |  |
| Yes | 212 (92.98 %) | 16 (7.02 %) | 585 (81.14 %) | 136 (18.86 %) | 448 (67.88 %) | 212 (32.12 %) |
| No | 823 (97.98 %) | 17 (2.02 %) | 439 (87.98 %) | 60 (12.02 %) | 154 (71.63 %) | 61 (28.37 %) |
| **Coronary Artery Dis.** |  |  |  |  |  |  |
| Yes | 22 (84.62 %) | 4 (15.38 %) | 147 (80.77 %) | 35 (19.23 %) | 152 (63.6 %) | 87 (36.4 %) |
| No | 1013 (97.22 %) | 29 (2.78 %) | 877 (84.49 %) | 161 (15.51 %) | 450 (70.75 %) | 186 (29.25 %) |
| **Chronic Kidney Dis.** |  |  |  |  |  |  |
| Yes | 46 (88.46 %) | 6 (11.54 %) | 123 (75 %) | 41 (25 %) | 170 (63.91 %) | 96 (36.09 %) |
| No | 989 (97.34 %) | 27 (2.66 %) | 901 (85.32 %) | 155 (14.68 %) | 432 (70.94 %) | 177 (29.06 %) |
| **Chronic Liver Dis.** |  |  |  |  |  |  |
| Yes | 20 (95.24 %) | 1 (4.76 %) | 25 (71.43 %) | 10 (28.57 %) | 10 (55.56 %) | 8 (44.44 %) |
| No | 1015 (96.94 %) | 32 (3.06 %) | 999 (84.3 %) | 186 (15.7 %) | 592 (69.08 %) | 265 (30.92 %) |
| **Liver Cirrhosis** |  |  |  |  |  |  |
| Yes | 6 (100 %) | 0 (0 %) | 9 (60 %) | 6 (40 %) | 3 (50 %) | 3 (50 %) |
| No | 1029 (96.89 %) | 33 (3.11 %) | 1015 (84.23 %) | 190 (15.77 %) | 599 (68.93 %) | 270 (31.07 %) |

**Suppl. Table 5.** Multivariable relationships of selected anthropometrics, comorbidities and laboratory parameters with COVID-19-related mortality in 3 age groups

| **Young/Middle-aged Older Old** | | | | | | |
| --- | --- | --- | --- | --- | --- | --- |
| **Characteristics** | **OR** | **p** | **OR** | **p** | **OR** | **P** |
| Sex female (ref) |  |  |  |  |  |  |
| Sex male | 3.07 | 0.027 | 1.27 | 0.18 | 1.58 | 0.0031 |
| BMI 18.5 - 24.9 (ref) |  |  |  |  |  |  |
| BMI 25 - 29.9 | 1.94 | 0.24 | 0.99 | 0.98 | 0.92 | 0.61 |
| BMI 30 - 34.9 | 2.53 | 0.12 | 0.90 | 0.67 | 0.93 | 0.73 |
| BMI ≥35 | 4.67 | 0.013 | 2.02 | 0.0059 | 0.57 | 0.16 |
| No Diabetes (ref) |  |  |  |  |  |  |
| Diabetes | 1.66 | 0.39 | 1.41 | 0.11 | 1.48 | 0.042 |
| HbA1c <6.4% (ref) |  |  |  |  |  |  |
| HbA1c 6.4% - 8 % | 0.92 | 0.95 | 4.72 | 0.15 | 2.14 | 0.23 |
| HbA1c 8.1% - 10% | 1.09 | 0.96 | 4.91 | 0.16 | 2.97 | 0.13 |
| HbA1c >10% | 1.11 | 0.95 | 19.5 | 0.0079 | 2.62 | 0.35 |
| HbA1c unknown | 0.93 | 0.95 | 10.1 | 0.027 | 3.77 | 0.022 |
| No Hypertension (ref) |  |  |  |  |  |  |
| Hypertension | 1.73 | 0.20 | 1.46 | 0.038 | 1.16 | 0.42 |
| No Coronary Artery Dis. (ref) |  |  |  |  |  |  |
| Coronary Artery Dis. | 2.60 | 0.15 | 1.13 | 0.58 | 1.19 | 0.29 |
| No Chronic Kidney Dis. (ref) |  |  |  |  |  |  |
| Chronic Kidney Dis. | 2.37 | 0.15 | 1.75 | 0.0091 | 1.32 | 0.089 |
| No Liver Cirrhosis (ref) |  |  |  |  |  |  |
| Liver Cirrhosis | 0.00 | 0.99 | 3.35 | 0.029 | 1.58 | 0.58 |

**Suppl. Table 6.** Multivariable relationships of selected anthropometrics, comorbidities and laboratory parameters with severity of COVID-19 in 3 age groups

| **Characteristics OR Lower Upper p**  **95%CI 95%CI** | | | | |
| --- | --- | --- | --- | --- |
| Young/Middle-aged - no Obesity, no Diabetes, no Hypertension (ref.) (N=593) |  |  |  |  |
| Young/Middle-age - Obesity, no Diabetes, no Hypertension (N=195) | 2.28 | 0.95 | 5.50 | 0.06 |
| Young/Middle-aged - Obesity, Diabetes, no Hypertension (N=24) | 5.34 | 2.33 | 13.5 | 0.0002 |
| Young/Middle-aged - Obesity, Diabetes, Hypertension (N=31) | 2.60 | 1.87 | 3.64 | <0.0001 |
| Older - no Obesity, no Diabetes, no Hypertension (N=339) | 2.66 | 2.01 | 3.52 | <0.0001 |
| Older - Obesity, no Diabetes, no Hypertension (N=92) | 16.02 | 5.16 | 71.0 | <0.0001 |
| Older - Obesity, Diabetes, no Hypertension (N=28) | 8.45 | 5.03 | 14.7 | <0.0001 |
| Older - Obesity, Diabetes, Hypertension (N=148) | 4.26 | 2.68 | 6.88 | <0.0001 |
| Old - no Obesity, no Diabetes, no Hypertension (N=166) | 5.41 | 3.67 | 8.07 | <0.0001 |
| Old - Obesity, no Diabetes, no Hypertension (N=25) | 4.11 | 0.85 | 29.3 | 0.10 |
| Old - Obesity, Diabetes, no Hypertension (N=7) | 4.84 | 2.65 | 9.07 | <0.0001 |
| Old - Obesity, Diabetes, Hypertension (N=80) | 12.32 | 4.55 | 43.0 | <0.0001 |
| Sex Male | 1.56 | 1.27 | 1.94 | <0.0001 |
| HbA1c 6.4% - 8% | 1.68 | 0.50 | 5.21 | 0.38 |
| HbA1c 8.1% - 10% | 1.81 | 0.49 | 6.49 | 0.36 |
| HbA1c >10% | 1.33 | 0.28 | 6.78 | 0.72 |
| HbA1c unknown | 1.28 | 0.41 | 3.53 | 0.65 |
| Coronary Artery Disease | 1.01 | 0.66 | 1.56 | 0.97 |
| Chronic Kidney Disease | 1.63 | 1.08 | 2.49 | 0.02 |
| Liver Cirrhosis | 0.34 | 0.10 | 1.05 | 0.07 |

**Suppl. Table 7.** Multivariable relationships of four age groups based on the presence (unhealthy) or absence (healthy) of obesity, diabetes and hypertension and selected anthropometrics, comorbidities and laboratory parameters with COVID-19-related mortality

| **Model 1**  **Characteristics OR Lower Upper p**  **95%CI 95%CI** | | | | |
| --- | --- | --- | --- | --- |
| Young - no Obesity, no Diabetes, no Hypertension (ref.) (N=203) |  |  |  |  |
| Young - Obesity, no Diabetes, no Hypertension (N=50) | 0.0003 | - | - | 0.98 |
| Young - Obesity, Diabetes, no Hypertension (N=1) | 211 | 5.80 | 10741 | 0.002 |
| Young - Obesity, Diabetes, Hypertension (N=2) | 4.24 | 0.17 | 108 | 0.31 |
| Middle-aged - no Obesity, no Diabetes, no Hypertension (ref.) (N=390) | 4.12 | 0.75 | 76.7 | 0.18 |
| Middle-age - Obesity, no Diabetes, no Hypertension (N=145) | 9.32 | 0.36 | 244 | 0.12 |
| Middle-aged - Obesity, Diabetes, no Hypertension (N=23) | 14.34 | 1.31 | 319 | 0.03 |
| Middle-aged - Obesity, Diabetes, Hypertension (N=29) | 5.72 | 0.84 | 113 | 0.12 |
| Older - no Obesity, no Diabetes, no Hypertension (N=339) | 25.2 | 5.40 | 449 | 0.002 |
| Older - Obesity, no Diabetes, no Hypertension (N=92) | 40.2 | 5.82 | 804 | 0.001 |
| Older - Obesity, Diabetes, no Hypertension (N=28) | 54.2 | 11.0 | 981 | 0.0001 |
| Older - Obesity, Diabetes, Hypertension (N=148) | 23.5 | 4.39 | 435 | 0.003 |
| Old - no Obesity, no Diabetes, no Hypertension (N=166) | 74.4 | 15.87 | 1328 | <0.0001 |
| Old - Obesity, no Diabetes, no Hypertension (N=25) | 22.7 | 0.82 | 633 | 0.04 |
| Old - Obesity, Diabetes, no Hypertension (N=7) | 85.3 | 16.7 | 1562 | <0.0001 |
| Old - Obesity, Diabetes, Hypertension (N=80) | 90.4 | 15.16 | 1,735. | <0.0001 |
| Sex Male | 1.37 | 0.98 | 1.95 | 0.07 |
| HbA1c 6.4% - 8% | 1.41 | 0.38 | 6.82 | 0.63 |
| HbA1c 8.1% - 10% | 1.99 | 0.50 | 10.1 | 0.36 |
| HbA1c >10% | 3.47 | 0.67 | 20.8 | 0.15 |
| HbA1c unknown | 2.28 | 0.70 | 10.3 | 0.22 |
| Coronary Artery Disease | 1.13 | 0.70 | 1.79 | 0.60 |
| Chronic Kidney Disease | 1.77 | 1.16 | 2.69 | 0.008 |
| Liver Cirrhosis | 1.55 | 0.32 | 5.63 | 0.54 |

OR, odds ratio; CI, confidence interval; Model 1, adjusted for sex, HbA1c, coronary artery disease, chronic kidney disease and liver cirrhosis

**Suppl. Figure 1.** Study flow chart

**
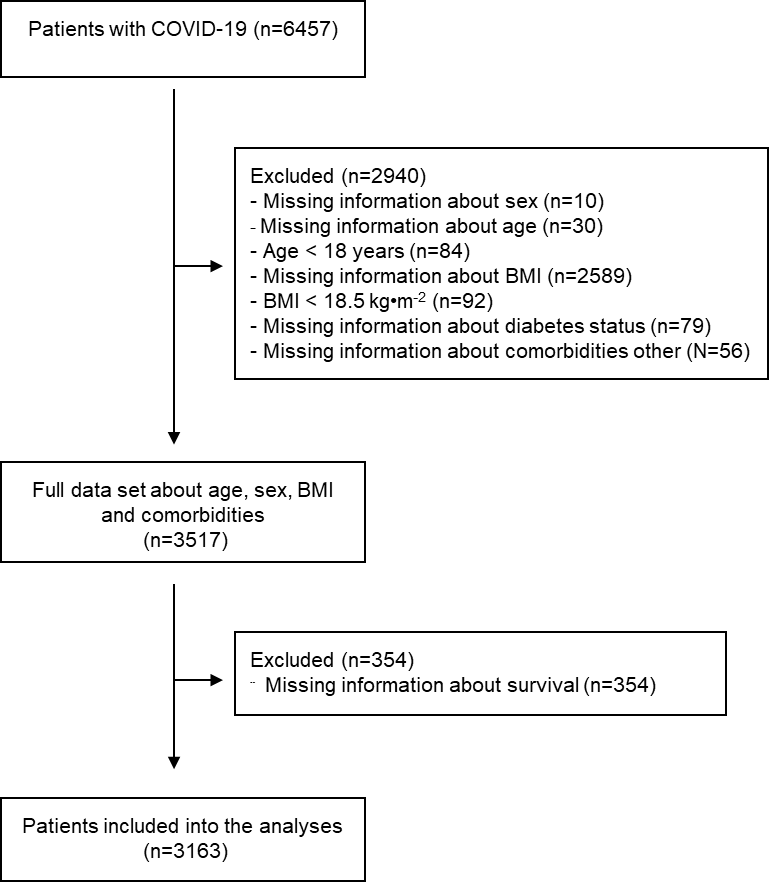
**

**Suppl. Figure 2.** Multivariable relationships of selected anthropometrics, comorbidities and laboratory parameters with COVID-19-related mortality


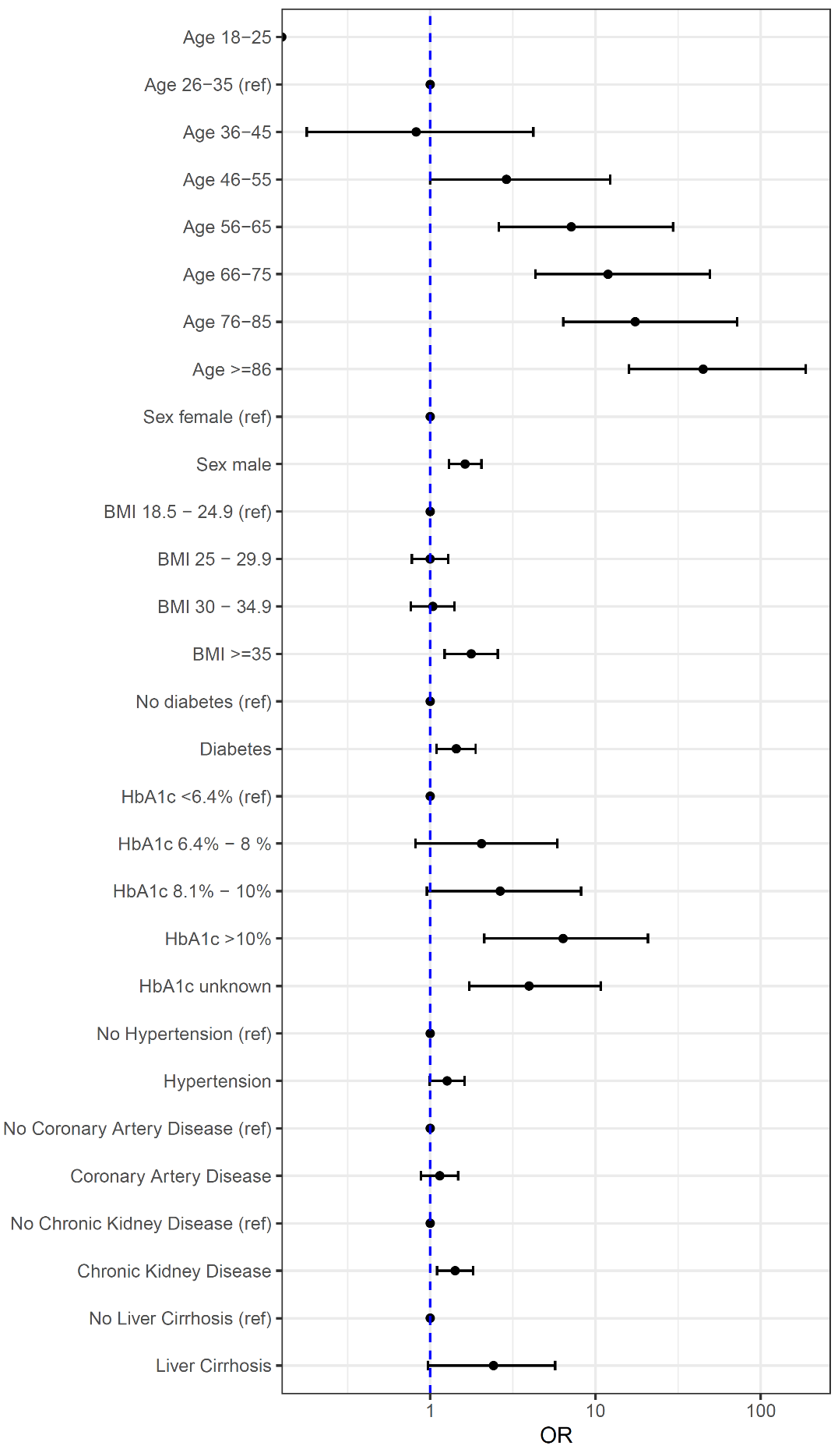


**Legends of the supplemental figures**

**Suppl. Figure 1**

Study flow chart.

**Suppl. Figure 2**

Adjusted odds ratios and 95% confidence intervals of anthropometrics, sex, comorbidities and laboratory parameters with COVID-19-related mortality in 3163 patients. All parameters shown were included in the multivariate regression analysis.
